# New leadership for WHO Western Pacific region: a call to prioritize oral health in the Pacific Islands

**DOI:** 10.3389/fpubh.2024.1388117

**Published:** 2024-07-08

**Authors:** Leenu Maimanuku, Susitina Piukala, Leroy Tatui, Kantara Tiim, Habib Benzian

**Affiliations:** ^1^School of Dentistry and Oral Health, College of Medical and Health Sciences, Fiji National University, Suva, Fiji; ^2^Ministry of Health, Apia, Tonga; ^3^Department of Health, Alofi, Niue; ^4^Department Epidemiology and Health Promotion, WHO Collaborating Centre Quality Improvement & Evidence-based Dentistry, College of Dentistry, New York University, New York, NY, United States

**Keywords:** oral health, public policy, regional health agency, World Health Organization (WHO), policy priority, NCD and risk factors

## What we already know

- Oral diseases are major public health problems, affecting more than 800 million people in the WHO Western Pacific region.
- More than half of the region's member states have no oral health policy, and more than a quarter have no dedicated staff for oral health in their health ministries.
- Pacific Island countries face significant barriers to oral disease prevention and care due to isolation, resource and infrastructure gaps, and limited oral health workforce.

## What this article adds

- Stresses the need for higher prioritization of oral health, particularly in the Pacific Islands.
- Introduces the “Suva Declaration on Improving Oral Health in the Pacific Islands Region” as a blueprint to develop recommendations and action on oral health within WHO WPR.
- Advocates for integrating oral health in the regional policy agenda to enhance Primary Health Care and advance Universal Health Coverage efforts.

## Introduction

The World Health Organization's Western Pacific Region (WHO WPR) has elected a new regional director, Dr. Saia Ma'u Piukala, a public health physician and Minister of Health from Tonga (1). The first nomination of a leader from the Pacific Islands Region since the organization's foundation in 1948 marks a seismic shift for the organization. Dr. Piukala's election also comes at a time when the WHO is navigating both internal reform initiatives and a variety of external challenges, making his fresh perspective potentially transformative for the region among major geo-political tensions (2).

WHO WPR spans over 37 diverse countries and territories, from densely populated megacities to small island developing states with minuscule populations, including a spectrum ranging from fragile least-developed economies to high-income nations. Non-communicable diseases (NCDs) are a priority health challenge, with Pacific Island countries experiencing some of the highest NCD-related morbidity and mortality globally (3). Contributing factors such as tobacco and alcohol use, unhealthy diets, and negative aspects of globalization have also led to a significant burden of oral diseases, a subset of NCDs that has been underprioritized in WHO WPR's recent political agenda (4).

## Oral disease challenges in the WHO Western Pacific region

The slate of regional oral health challenges is huge and has reached unprecedented levels. Eight hundred million people suffer from oral diseases such as dental caries, severe periodontal disease, oral cancer, or edentulism, as highlighted by last year's regional summary of the WHO Global Oral Health Status (5). The report also showed a higher burden for most diseases in populations of Pacific Island countries, including some of the highest rates of oral cancer globally.

The alarmingly high burden of oral disease and the resulting negative consequences for health, education, social life, economic productivity, and wellbeing should be a wake-up call for all stakeholders. There is an urgent need to step up policy support and political leadership, together with increased investments in an appropriate oral health workforce, functioning infrastructure, and the availability of essential dental medicines and supplies. Oral healthcare grapples with significant shortages of trained professionals and under-resourced public services, which provide most of the oral healthcare services in Pacific Island countries. With more than half of the region's countries spending < $10 USD per person per year on oral health, governments need to step up their investments and efforts to strengthen oral healthcare infrastructure, workforce, and quality in the context of primary healthcare and Universal Health Coverage (UHC). Additionally, there is often scarce or no access to oral healthcare for rural, remote, or socioeconomically disadvantaged communities.

Yet, more than half of the region's countries have no oral health policy, and more than a quarter have no dedicated staff at the Ministry of Health to support planning, service delivery, monitoring, and evaluation for oral diseases. The WHO Global Strategy on Oral Health and the Global Oral Health Action Plan provide crucial guidance and leadership (6, 7), but WHO WPR is lagging behind other WHO regions in developing regional guidance and technical support for countries.

## Strengthening oral health systems with a focus on Pacific Island countries

In response to the region's oral health needs, a coalition of heads of oral health services established the Oral Health Pacific Islands Alliance (OPIA) (8). At a meeting in Suva (Fiji) in 2014, they developed the *Suva Declaration on Improving Oral Health in the Pacific Islands Region*, a technical and policy consensus document to advocate for better recognition and access to oral healthcare. An evaluation almost 10 years later revealed patchy progress across the region and identified health system shortcomings requiring urgent attention. The Suva Declaration was reconfirmed in 2023, including amended key recommendations to align with the new WHO Global Oral Health Action Plan adopted in May 2023 (Table 1) (9).

**Table 1:** The Suva Declaration on Improving Oral Health in the Pacific Islands Region - priority commitments aligned with the WHO Global Oral Health Action Plan.

**Strategic objective 1—Governance**
Review existing or develop new national oral health policies integrated with relevant NCD and UHC planning frameworks by 2025
Focus on building and strengthening health service capacities to address oral diseases by expanding and broadening of the oral health workforce, fostering competencies in public health and planning, as well as leadership and succession planning
**Strategic objective 2—Oral health promotion and prevention**
Advocate for inclusion of essential oral medicines in national essential medicines lists and to improve their affordability through appropriate measures, such as bulk purchasing and fiscal measures by 2030
Develop, review, and evaluate new and existing community-based oral health promotion programmes along the life course, such as integrated mother-child health, school health, workplace, special needs and aged care, by 2025
**Strategic objective 3—Oral health workforce**
Review the existing and establish new integrated national workforce plans that include the oral health workforce by 2025
Reconfirm and align with all measures outlined in the Suva Declaration related to oral health workforce and strengthen action on them
**Strategic objective 4—Oral health care**
Advocate for countries to become parties of the Minamata Convention on Mercury and to develop a national plan to phase-out the use of dental amalgam by 2029
Commit digital health resources to strengthen oral health service delivery by 2025
Integrate essential oral health services as part of primary health care and Universal Health Coverage
**Strategic objective 5—Oral health information**
Advocate for integration of oral health information in the Healthy Islands Monitoring Framework
Reconfirm and align with all measures related to oral health information outlined in the Suva Declaration and strengthen action on them
**Strategic objective 6—Oral health research**
Strengthen research through pragmatic data collection, analysis, and impact evaluation of programmes and policies, and evidence-generation to inform decision making and planning at all levels
Develop national oral health research priorities and a plan on how to address them by 2025
Promote and seek research opportunities and partnerships to enhance regional research capacities and translation into action

The Suva Declaration on Improving Oral Health in the Pacific Islands Region was developed in 2014 and reconfirmed in 2023 during a meeting of heads of oral health services and other stakeholders from 13 countries of the region (9). They amended the Suva Declaration with commitments for accelerated action on oral diseases based on the six action areas of the WHO Global Oral Health Action Plan.

The full text of the Suva Declaration is available at https://bit.ly/suva-declaration-2023 or scan here:

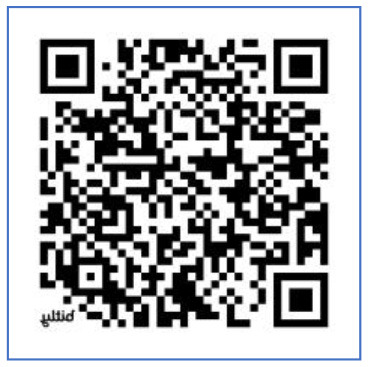

As the new WHO WPR Regional Director steps into his role, defining and framing his priorities becomes critical. His predecessor prioritized NCDs, aging, and climate change (10). We urge WHO WPR to recognize the urgency of impactful measures against oral diseases, particularly in the Pacific Island region with its distinct problems, guided by the technical consensus recommendations of the Suva Declaration.

## Conclusion

To provide effective oral health guidance and support for member states, the current WHO WPR deficits in policy leadership and technical capacities need to be addressed. The appointment of a regional oral health adviser would mark an essential step in enhancing WPRO's expertise in integrating oral health within the broader context of NCDs and UHC. Advancing discussions at the regional committee level toward a resolution on oral health would foster alignment and encourage action guided by the WHO Global Oral Health Action Plan. Collaborative efforts with stakeholders such as OPIA, the Pacific Community, WHO Collaborating Centers, and universities such as Fiji National University, the only regional oral health training institution, will be instrumental in addressing the oral health challenges in Pacific Island countries and across all WPR member states.

## Author contributions

LM: Conceptualization, Resources, Writing – review & editing. SP: Conceptualization, Resources, Writing – review & editing. LT: Conceptualization, Resources, Writing – review & editing. KT: Conceptualization, Resources, Writing – review & editing. HB: Conceptualization, Project administration, Writing – original draft, Writing – review & editing.

## References

[1] Dr Saia Ma'u Piukala appointed WHO Regional Director for the Western Pacific. In: WHO WPRO News Release 23 January 2024. (2024). Available online at: https://www.who.int/westernpacific/news/item/23-01-2024-dr-saia-ma-u-piukala-appointed-who-regional-director-for-the-western-pacific (accessed July 1, 2024).

[2] Kapila M. Geopolitics at heart of elections for new director of WHO Western Pacific Region. In: Health Policy Watch. (2023). Available online at: https://healthpolicy-watch.news/geopolitics-at-heart-of-elections-for-new-director-of-who-western-pacific-region/ (accessed July 1, 2024).

[3] Peng W Zhang L Wen F Tang X Zeng L Chen J. Trends and disparities in non-communicable diseases in the Western Pacific region. Lancet. (2023) 43:100938. 10.1016/j.lanwpc.2023.100938 38456093 PMC10920054

[4] Pili N Nosa V Tatui L. Implementing policies and programmes to reduce the impact of globalisation on oral health in Pacific Island Countries and Territories. J Global Health Econ Policy. (2021) 1:29655. 10.52872/001c.29655

[5] World Health Organization (WHO). Global Oral Health Status Report - Towards Universal Health Coverage for Oral Health 2030. In: Regional Summary of the Western Pacific Region. Geneva: WHO (2023). Available online at: https://www.who.int/publications/i/item/9789240070868 (accessed July 1, 2024).

[6] World Health Organization (WHO). Draft Global Strategy on Oral Health (WHO EB150/7 Annex 3). Geneva: WHO (2022). Available online at: https://apps.who.int/gb/ebwha/pdf_files/EB150/B150_7-en.pdf (accessed July 1, 2024).

[7] World Health Organization (WHO). Global Oral Health Action Plan (2023–2030). Available online at: https://www.who.int/publications/i/item/9789240090538 (accessed July 1, 2024).

[8] Tatui L McCool J Nosa V. Rethinking and establishing a dental collaboration in the Pacific region. Pacific Health Dialog. (2018) 21:108–110. 10.26635/phd.2018.921

[9] Oral Health Pacific Islands Alliance (OPIA). Suva Declaration on Improving Oral Health in the Pacific Islands Region. (2023). Available online at: https://www.fnu.ac.fj/wp-content/uploads/2023/08/Suva-Declaration-2023.pdf (accessed July 1, 2024).

[10] Kasai T. For the future: delivering better health in the Western Pacific region. J Global Health Sci. (2019) 1:e27. 10.35500/jghs.2019.1.e27

